# Platelet inhibitory effects of juices from *Pachyrhizus erosus* L. root and *Psidium guajava* L. fruit: a randomized controlled trial in healthy volunteers

**DOI:** 10.1186/s12906-016-1255-1

**Published:** 2016-08-03

**Authors:** Thitiporn Thaptimthong, Thitima Kasemsuk, Nathawut Sibmooh, Supeenun Unchern

**Affiliations:** Department of Pharmacology, Faculty of Science, Mahidol University, Rama VI Road, Bangkok, 10400 Thailand

**Keywords:** Yam bean root juice, Guava fruit juice, Systemic nitrate, Systemic nitrite, Plasma cGMP, Antiplatelet effect, Blood pressure lowering effect

## Abstract

**Background:**

The purpose of this study is to investigate cardiovascular benefits of juices obtained from two commonly consumed fruits in Thailand, *Pachyrhizus erosus*, L. (yam bean) and *Psidium guajava*, L. (guava), by examining their acute cardiovascular effects in healthy volunteers. Possible involvements of the dietary nitrate on their effects were investigated as well.

**Method:**

Thirty healthy volunteers were randomly divided into three groups of 10 subjects per group and each group was allocated to drink 500 ml of freshly prepared yam bean root juice, guava fruit juice, or water. Systemic nitrate and nitrite concentrations, heart rate, systolic and diastolic blood pressure, serum K^+^ concentrations, ex vivo platelet aggregation, and plasma cGMP concentrations were monitored at the baseline and at various time points after the intake of juices or water. Data were compared by repeated measures ANOVA.

**Results:**

Following the ingestion of both yam bean root juice and guava fruit juice, collagen-induced but not ADP-induced platelet aggregation was attenuated. Ingestion of yam bean root juice increased systemic nitrate and nitrite concentrations whereby elevated nitrite concentrations correlated with the extent of inhibiting collagen-induced platelet aggregation. In addition, positive correlation between systemic nitrite and plasma cGMP concentrations and negative correlation between plasma cGMP concentrations and the extent of collagen-induced platelet aggregation were revealed. Nevertheless, yam bean root juice reduced only diastolic blood pressure while guava fruit juice reduced heart rate, systolic and diastolic blood pressure.

**Conclusion:**

The present study has illustrated, for the first time, acute inhibitory effects of yam bean root juice and guava fruit juice on ex vivo collagen-induced platelet aggregation in healthy subjects. Dietary nitrate was shown to underlie the effect of yam bean root juice but not that of guava fruit juice. Following yam bean root juice ingestion, systemic nitrate apparently converts to nitrite and further to NO which may attenuate platelet responses to collagen stimulation. Cardiovascular benefits of juices from yam bean root and guava fruit are noteworthy in term of the cardiovascular health-promoting approach.

**Trial registration:**

Randomized controlled trial TCTR20150228001.

## Background

Decreased production of nitric oxide (NO) is evident in essential hypertension and other conditions associated with elevated blood pressure such as diabetes, hypercholesterolemia, and chronic kidney disease [1–6]. Evidence from both human and animal studies showed that nitrate and nitrite derived from diet can serve as a source for NO particularly where it is deficient [7–10], which was firstly demonstrated by Appel and coworkers in 1997 [9]. Nitrate rich diets including green leafy and cruciferous vegetables were reported to have a protective effect on cardiovascular diseases [11, 12].

Following the oral administration of sodium nitrate to healthy volunteers, a significant fall in diastolic and mean arterial blood pressure in association with increasing plasma nitrate was previously demonstrated [13]. Beetroot, a vegetable with high organic nitrate content, reduced blood pressure of healthy volunteers, an effect that correlated with peak increases in plasma nitrite concentrations [14]. Intake of beetroot juice also significantly attenuated ex vivo ADP- and collagen-induced platelet aggregation [14]. In addition, it was demonstrated that the reduction of nitrate to nitrite by oral commensal bacteria was required for blood pressure lowering and platelet inhibitory effects [14].

The formation of blood clot in the circulatory system can lead to disturbances in the blood supply resulting in embolism and stroke. Several fruit juices seem to be able to limit blood clot formation by preventing platelet aggregation in blood vessels [15, 16]. Fruit juices also play a role in the maintenance of NO levels. This has been shown to occur with many juices [17] including grape [18], pomegranate [19], and citrus juices [20, 21]. These beneficial changes have been noted at various aspects of cardiovascular system including blood pressure [20, 22]. In the prevention of cardiovascular diseases, the consumption of fruits and vegetables is crucial.

Guava (*Psidium guajava* L.) belongs to the family Myrtaceae and is a traditionally used plant in tropical and subtropical countries because of its food and nutritional value. Every part of guava plant including leaves, root, bark, and fruits has been used for medicinal purposes [23]. The vasodilator and antioxidant actions exerted by guava extracts have been reported [24]. Antihypertensive effect of guava fruit (GF) on spontaneous hypertensive rats was previously demonstrated [25]. Studies using guava leaf extract showed cardio-protective effects against myocardial ischemia-reperfusion injury in the isolated rat heart [26] and cardio-inhibitory action in rats and guinea pig models [27].

Yam bean (*Pachyrhizus erosus* L.) belongs to the family Leguminosae, subfamily Papilionoidea. In Thailand, yam bean’s tuberous root is consumed as fruit or vegetable. There was limited study on pharmacological effects of YBR. However, the crude fiber extract of YBR was shown to enhance the production of IgM, IgG, IgA, IL-5 and IL-10 by mouse splenocytes [28]. However, there is a lack of information regarding the effects of these plant juices on cardiovascular and platelet functions.

In Thailand, various kinds of fruits are available, however; for public health concern, guava fruit and yam bean root are appropriate for evaluating cardiovascular benefits because they are available throughout the year, not expensive, and thus consumable by all population. The present study was designed to investigate effects of ingesting juices from YBR and GF on heart rate (HR), systolic blood pressure (SBP), diastolic blood pressure (DBP), and platelet aggregation in healthy volunteers. We also examined the possibility that increased plasma nitrate can be achieved through the consumption of these juices, which may consequentially lead to acute reduction of blood pressure and heart rate, and inhibition of platelet aggregation, as a result of bioconversion to nitrite and NO in the body.

## Methods

### Chemicals

Adenosine diphosphate (ADP) and other chemicals were purchased from Sigma (St Louis, MO). Chrono Par (collagen) was purchased from Chrono-Log Corporation (Havertown, PA). Cyclic GMP ELISA kit was purchased from Assay Designs (Enzo Life Sciences, Inc., NY). Collagen, nitrite preservation solution, sodium nitrite, sodium nitrate, and triiodide solution were prepared freshly prior to use. ADP was dissolved in deionized water as 10 mmole/L stock solution and kept at −20 °C.

### Fruit materials and preparation of fruit juices

1. *Pachyrhizus erosus* L. (Common name: Yam bean; Thai name: Mun-kaew; Cultivar: Small root)
2. *Psidium guajava* L. (Common name: Guava; Thai name: Fa-rang; Cultivar: Pansetong)

Yam bean root (YBR) and guava fruit (GF) were purchased from Talaad Thai Market, Pathumthani Province, Thailand. Fruits were cleansed and juices were freshly prepared prior to the study by mechanical squeezing the fruit flesh with an electric juicer. Juices were then filtered to remove fibers and pulps. The percentage yield (% v/w) of juices from the fruit flesh was 47.62 % for YBR and 71.67 % for GF.

### Subjects

A sample size of 30 subjects completing the study was employed on the basis of estimates from our preliminary data. The sample size provided at least 95 % power to demonstrate a difference between mean of the treatment values. The study protocol was approved by the Clinical Research Ethics Committee of Ramathibodi Hospital, Mahidol University (protocol number: ID 03-54-25) that the study was conducted in compliance with the Declaration of Helsinki. Individual subject provided an informed consent after satisfying the inclusion criteria and understanding that he or she could withdraw at any time without a reason. The inclusion criteria were healthy volunteers aged between 20 to 45 years, body mass index (BMI) of 18–27 kg/m^2^, and singed informed consent. The exclusion criteria were a history of any serious illnesses, including infectious diseases, cardiovascular and pulmonary diseases, smoking and/or alcoholic drinking, systemic medication (other than oral contraceptive pills), and pregnancy.

### Study design

**Fig. 1:**
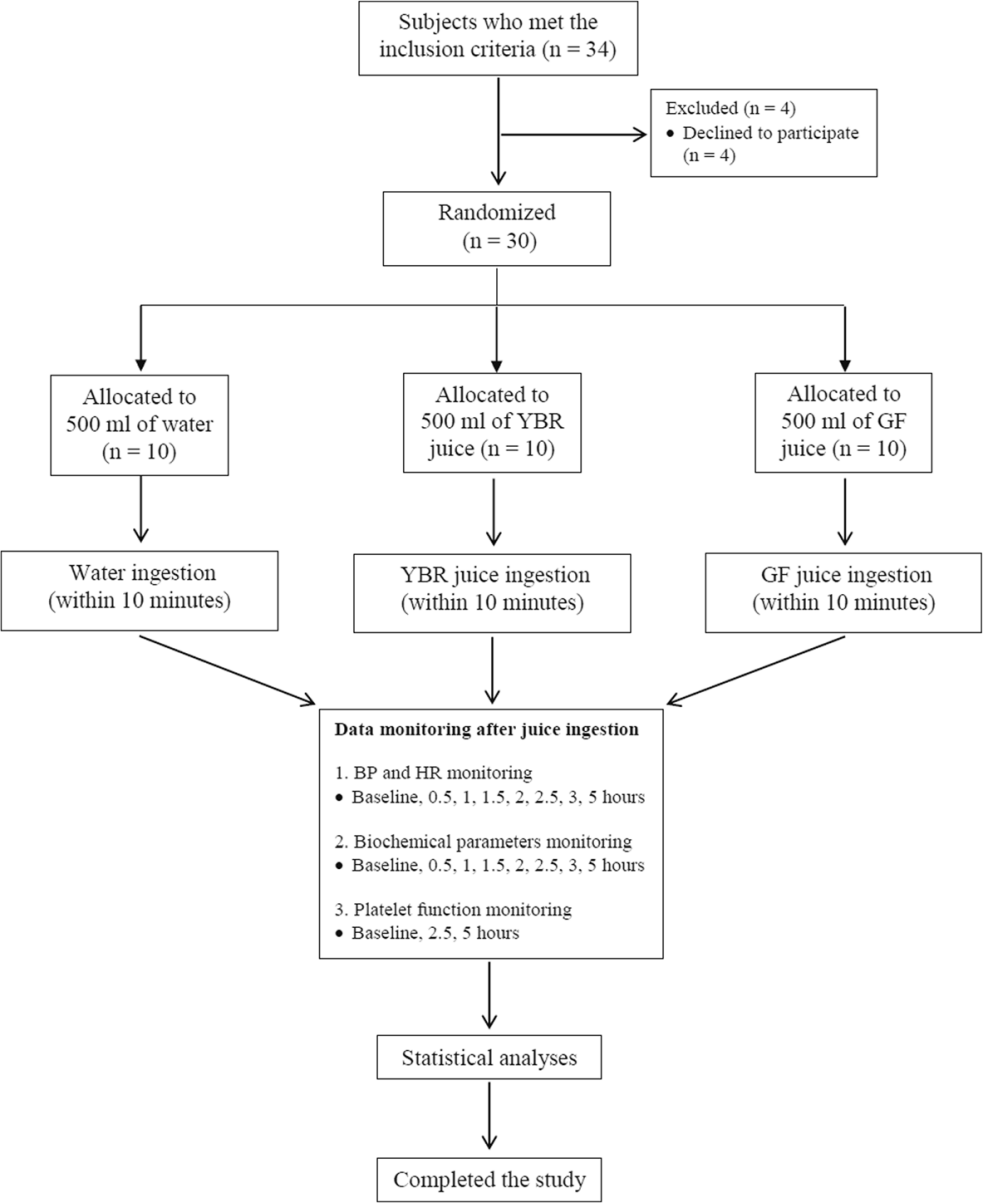
The flow diagram of progress through phases of the study. *Legend:* Data monitoring at the baseline was performed prior to the water or juice ingestion. (BP: blood pressure, HR: heart rate, YBR: yam bean root, GF: guava fruit)

The study protocol, an open-label, randomized block design, was summarized as a flow chart in Fig. 1. The 30 subjects were randomly allocated into 3 trial groups (*n* = 10 per group) to study the ingestion of 500 ml of YBR juice, GF juice or water. For the allocation of subjects, a drawing lots of subject list was used. Subjects were asked to stop taking certain medications or food supplements (e.g., NSAIDs, fish oil) for 14 days prior to the study, and avoid taking food with high nitrate contents such as processed meat or green leafy vegetables on a day preceding the study. Subjects were fasted overnight or at least 12 h prior to the studying day and during the morning of that particular day. On the studying day, all subjects were informed that the juice might cause a mild irritation to their stomach. The study took place at Pharmacological Laboratory, Department of Pharmacology, Faculty of Science, Mahidol University, under the supervision and care of physicians.

### Blood pressure measurement

Systolic blood pressure (SBP), diastolic blood pressure (DBP) and heart rate (HR) were measured at the baseline, and monitored every 30 min for 3 h, and then at 5 h following juices or water ingestion. All measurements were performed in triplicate with subjects in the seated position using an automatic blood pressure monitor (Omron, model T9P). The mean values of second and third readings were used for data analysis.

### Blood sampling

Ten milliters of blood samples were taken via a 22G intravenous catheter with extension set at the baseline and at 0.5, 1, 1.5, 2, 2.5, 3, and 5 h after juice or water ingestion. Blood samples were placed into lithium heparinized tubes and citrate tubes for the plasma preparation and assessment of platelet aggregation, respectively.

### Determination of nitrate concentrations

To determine systemic nitrate concentrations, blood samples were placed into heparinized tubes and gently mixed. The heparinized blood were centrifuged at 5,000 *g* at 4 °C for 1 min and then the plasma was separated and stored at −80 °C until the nitrate measurement. Nitrate concentrations were determined by a vanadium chloride-based chemiluminescence NO analyzer (CLD 88 sp, Eco Medics, Switzerland) using sodium nitrate as a standard [29]. Nitrate contents in juices and water were determined by the same method.

### Determination of nitrite concentrations

To determine systemic nitrite concentrations, blood samples were placed into heparinized tubes, gently mixed, and then centrifuged at 5,000 *g* for 1 min at 4 °C to separate plasma [30, 31]. All samples were stored at −80 °C until the nitrite measurement. Nitrite concentrations were determined by a triiodide-based chemiluminescence NO analyzer (CLD 88 sp, Eco Medics, Switzerland) using sodium nitrate as a standard [30]. Nitrite contents in juices and water were determined by the same method.

### Platelet aggregation study

Blood samples were collected from healthy volunteers and placed in plastic tubes containing 3.8 % sodium citrate in a ratio of 9:1 v/v. Platelet aggregation was assessed in platelet-rich plasma (PRP) which was prepared by centrifugation of citrated blood at 200 *g* for 10 min at 25 °C and the portion of platelet-rich supernatant was collected. The remaining portion was further centrifuged at 5,000 *g* for 10 min at 25 °C to obtain platelet-poor plasma (PPP). ADP (1–4 μmole/L) and collagen (0.5–2 μg/ml) were used as agonists for platelet aggregation. The PRP was incubated for 2 min at 37 °C, calibrated for 100 % light transmission against PPP, stirred for 1 min at a speed of 1,000 rpm prior to the addition of a platelet agonist (ADP or collagen). Platelet aggregation was monitored by a Chrono-Log aggregometer (Model 540 VS, Chronolog Corp., USA) using the optical aggregation technique as previously described [32, 33].

### Determination of plasma cGMP concentrations

Plasma cGMP levels were determined by using an enzyme immunoassay (Assay Designs' Correlate-EIA™ cyclic GMP kit) according to the manufacturer’s instruction. The heparinized blood samples were immediately centrifuged at 5,000 *g* for 1 min at 4 °C to separate plasma. Plasma samples were kept below −20 °C until the assay.

### Determination of serum potassium concentrations

Serum potassium ion levels were determined using an automated biochemical analyzer (Hitachi, model 912) which based on the ion-selective electrode technique [34].

### Statistical analysis

All data were analyzed using the Graph Pad Prism Software 5.01 and expressed as mean ± SEM. Data were compared by repeated measures ANOVA test followed by Dunnett’s posttest for comparison with baseline values, and followed by Bonferroni posttest for comparison with the control group. The value *P* < 0.05 was considered as statistically significant.

## Results and discussion

### Results

At the baseline, there was no significant difference in general characteristics among subjects in each group (Table 1). Both YBR and GF juices were palatable and well tolerated by all subjects. The mean (± SEM) concentrations of nitrate in YBR and GF juices were 5.231 (±0.173) and 0.355 (±0.030) mmole/L, respectively. However, nitrite contents of fruit juices and water were below the detection limit (<50 nmole/L) of the analytical assay.

**Table 1:** Demographic characteristics of healthy volunteers at the baseline

Characteristics	Water (control)	YBR juice	GF juice	Significance value
Subject (n)	10	10	10	*P* > 0.05
1. Male	3	3	4	*P* > 0.05
2. Female	7	7	6	*P* > 0.05
Age (year)	27.2 ± 2.4	26.0 ± 2.9	25.4 ± 2.2	*P* > 0.05
Height (cm)	163.67 ± 8.34	164.92 ± 7.83	166.11 ± 6.92	*P* > 0.05
Weight (kg)	62.04 ± 12.28	61.17 ± 9.88	63.11 ± 11.33	*P* > 0.05
BMI (kg/m^2^)	23.07 ± 3.75	22.45 ± 2.30	22.78 ± 3.37	*P* > 0.05
Platelet × 10^5^ (per ml of WB)	2.19 ± 0.55	2.21 ± 0.62	2.50 ± 0.39	*P* > 0.05
Plasma nitrate (μmole/L)	30.16 ± 4.31	36.43 ± 5.32	30.35 ± 1.78	*P* > 0.05
Plasma nitrite (nmole/L)	124.04 ± 9.31	106.87 ± 18.40	140.12 ± 2.11	*P* > 0.05
Plasma cGMP (nmole/L)	5.37 ± 0.50	5.613 ± 2.15	6.97 ± 2.50	*P* > 0.05
Serum K^+^ (mmole/L)	3.98 ± 0.132	4.09 ± 0.10	4.01 ± 0.08	*P* > 0.05

Data are expressed as mean ± SEM from subjects in each group. Baseline values were measured at time points prior to YBR juice, GR juice, or water ingestion. Statistical analysis was performed using unpaired Student’s *t*-test *vs*. the control

### Systemic nitrate, systemic nitrite, and serum K^+^ concentrations

**Fig. 2:**
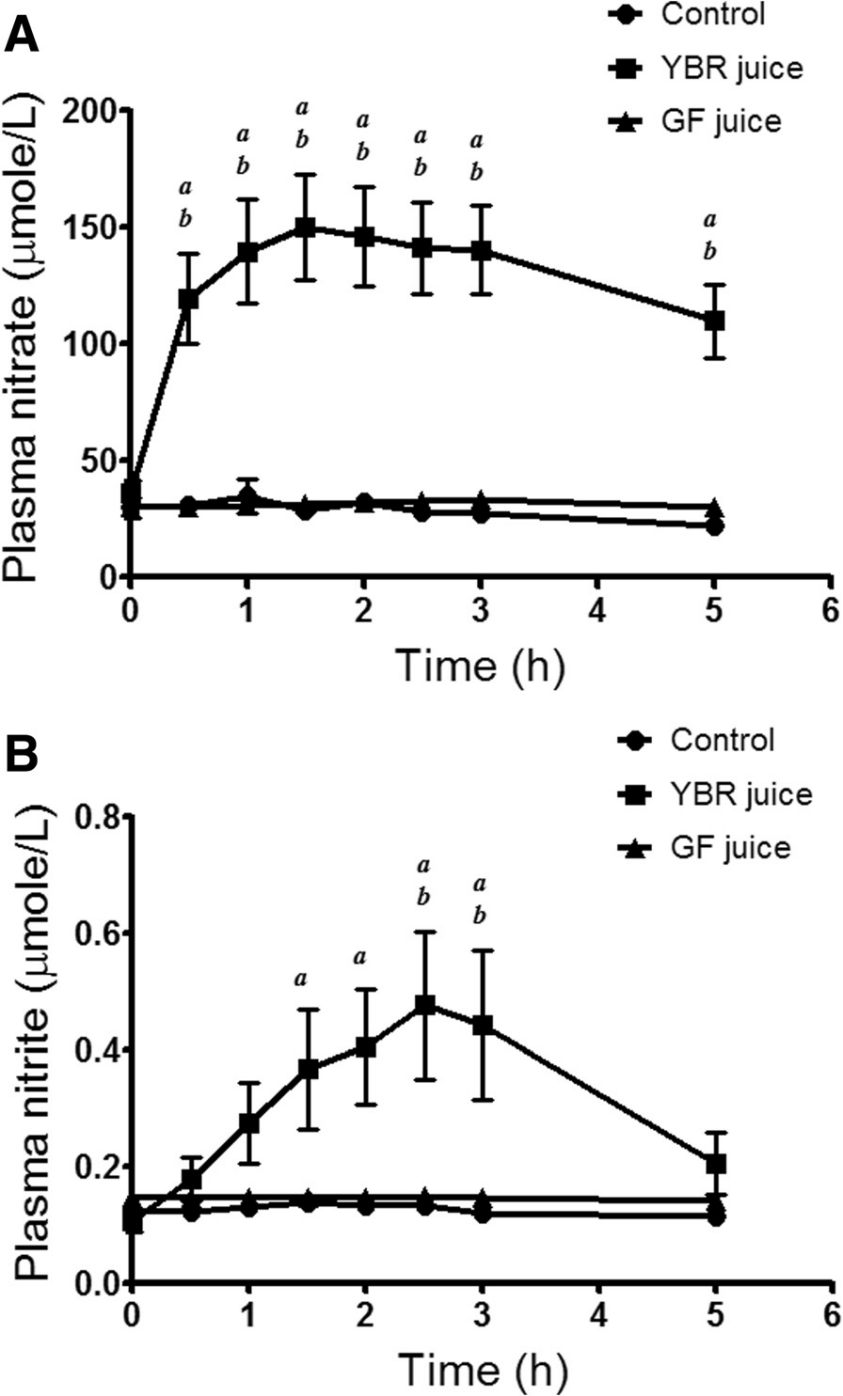
Time-plasma concentration profiles of nitrate and nitrite. *Legend*: Plasma nitrate (**a**) and plasma nitrite (**b**) concentrations in healthy volunteers at various time points after the ingestion of water, YBR juice, or GF juice. Data are expressed as mean ± SEM from 10 subjects in each group. ^*a*^ *P* < 0.05 Dunnett’s posttest compared to the baseline, ^*b*^ *P* < 0.05 Bonferroni posttest *vs.* the control

At the baseline, systemic nitrate concentrations in all subject groups were within the normal range, and there was no significant difference among them (Table 1). After YBR juice ingestion, as compared to the control group, there was a rapid and significant (*P <* 0.05) rise of systemic nitrate concentrations, starting within 30 min, peaking at 1.5 h and remaining at this level up to 5 h (Fig. 2a). However, there was no similar change after GF juice ingestion (Fig. 2a).

Baseline systemic nitrite concentrations of all subject groups were within the normal range (100–500 nmole/L) [35] and there was no significant difference among them (Table 1). Following YBR juice ingestion, as compared to the control group, systemic nitrite levels increased significantly (*P* < 0.05) after 1.5 h, peaking at 2.5 h (4.5 folds), remaining at this level up to 3 h then returned to near the baseline value by 5 h (Fig. 2b). There was no change in systemic nitrite concentrations after the ingestion of GF juice (Fig. 2b).

Baseline serum K^+^ concentrations of all subject groups were shown in Table 1. There was no significant difference among them. In addition, no significant alteration of serum K^+^ levels in healthy volunteers was apparent after the ingestion of YBR juice, GF juice, or water (data not shown).

### Effects on ex vivo platelet aggregation

**Fig. 3:**
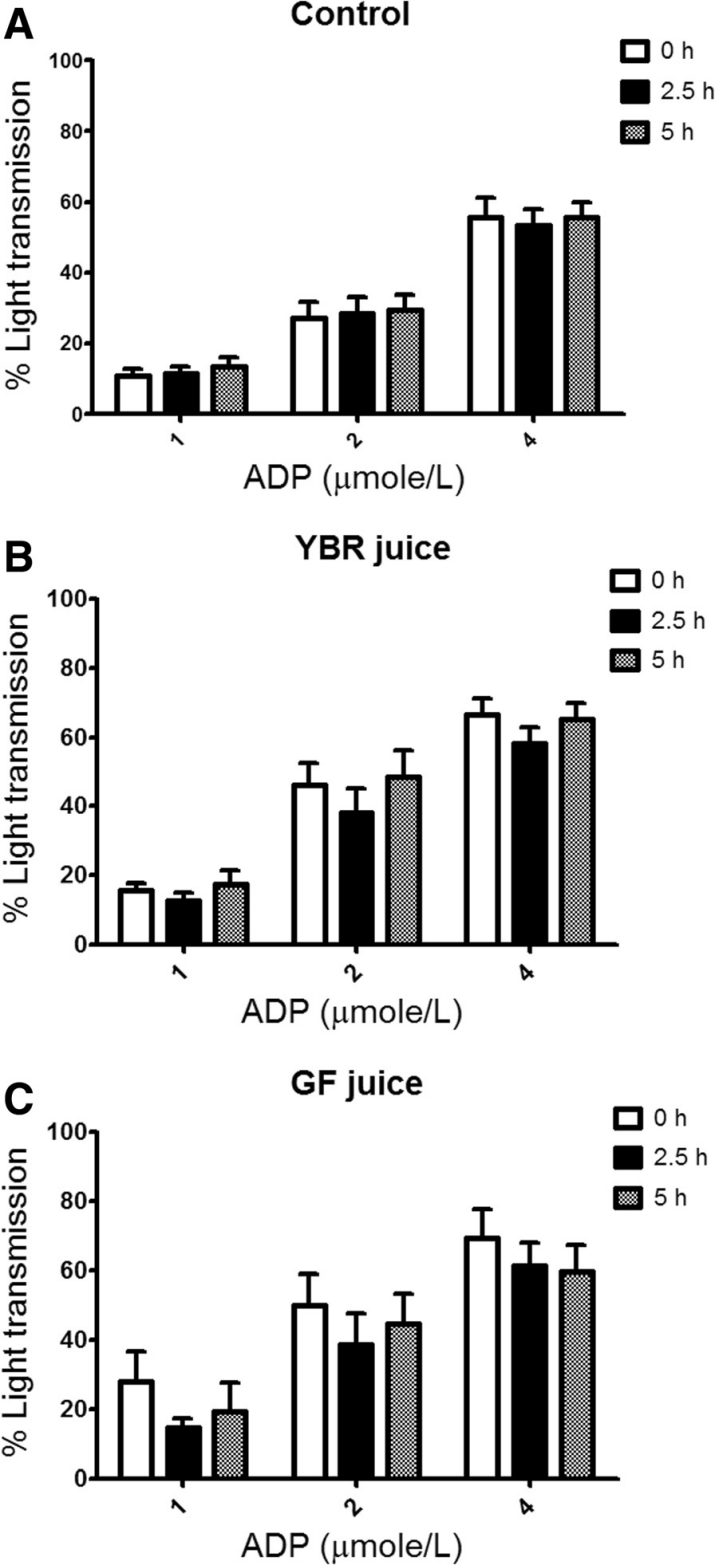
Effects of the juice ingestion on *ex vivo* ADP-induced platelet aggregation. *Legend: Ex vivo* ADP-induced platelet aggregation after the ingestion of water (**a**), YBR juice (**b**), or GF juice (**c**). Data are expressed as mean ± SEM from 10 subjects in each group. ^*a*^ *P* < 0.05 Dunnett’s posttest compared to the baseline

The mean platelet count of all subject groups was within the normal range (2–4 × 10^3^ per ml of the whole blood) and there was no significant difference among them (Table 1). At the baseline (t = 0 h), ADP dose-dependently induced the platelet aggregation with no difference in platelet responses among three subject groups (Fig. 3a, b and c). Both YBR and GF juices did not cause any significant alteration of the platelet aggregation induced by ADP (Fig. 3a, b and c).

**Fig. 4:**
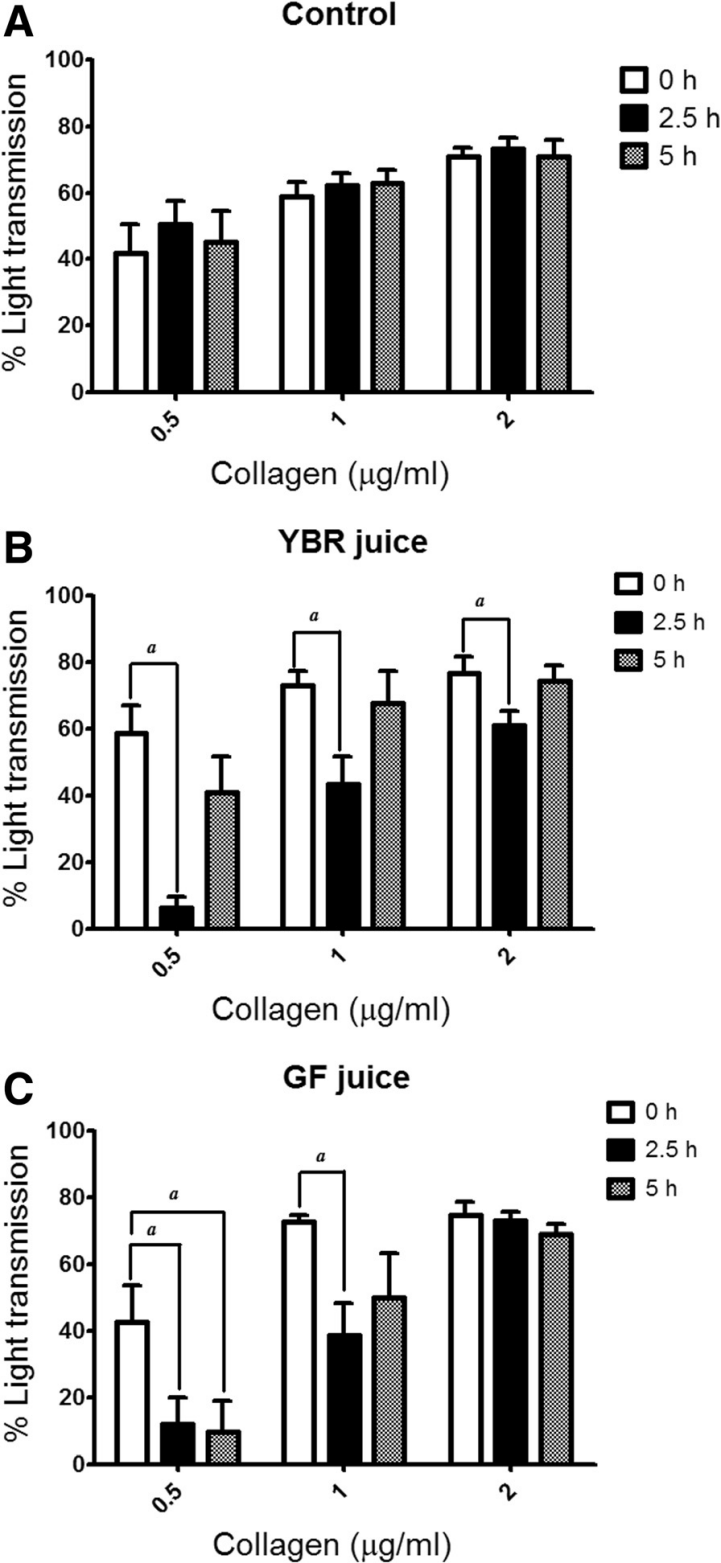
Effects of the juice ingestion on *ex vivo* collagen-induced platelet aggregation. *Legend: Ex vivo* collagen-induced platelet aggregation after the ingestion of water (**a**), YBR juice (**b**), or GF juice (**c**). Data are expressed as mean ± SEM from 10 subjects in each group. ^*a*^ *P* < 0.05 Dunnett’s posttest compared to the baseline

Collagen dose-dependently induced the platelet aggregation with no difference in platelet responses among three subject groups at the baseline (Fig. 4a, b and c). Following the ingestion of both YBR and GF juices, the ex vivo collagen-induced platelet aggregation was significantly attenuated (*P* < 0.05) as compared to that of control (Fig. 4a, b, and c).

### Effects on plasma cGMP concentrations

**Fig. 5:**
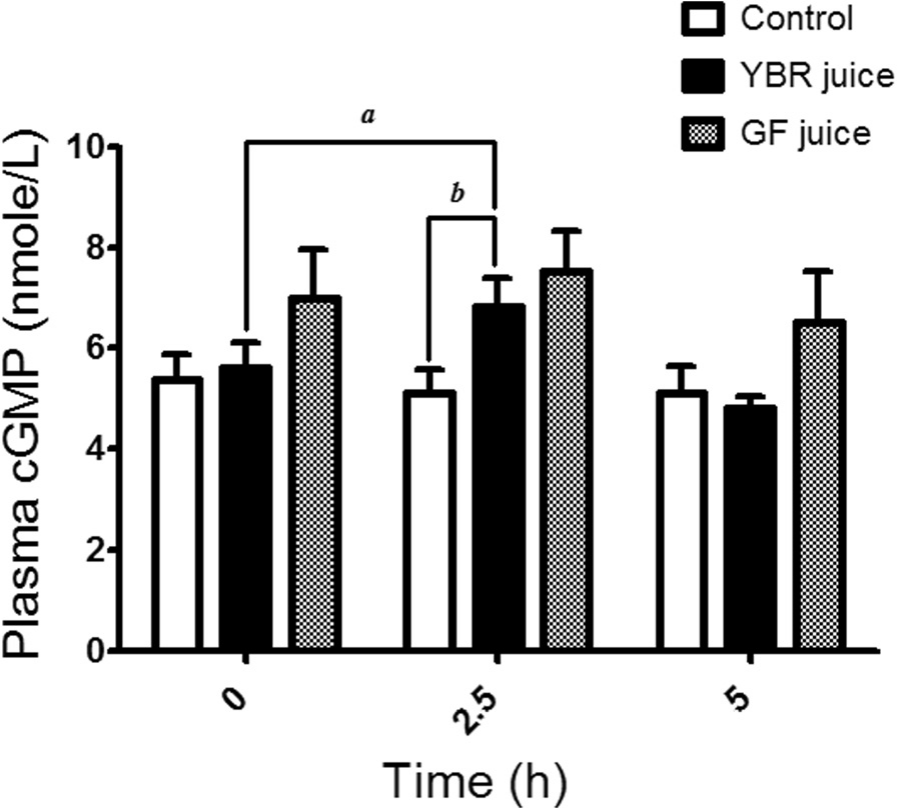
Effects of the juice ingestion on plasma cGMP concentrations. *Legend:* Plasma cGMP concentrations at three time points – baseline, 2.5 and 5 h after the ingestion of water, YBR juice, or GF juice. Data are expressed as mean ± SEM from 10 subjects in each group. ^*a*^ *P* < 0.05 Dunnett’s posttest compared to the baseline, ^*b*^ *P* < 0.05 Bonferroni posttest *vs.* the control

Plasma cGMP concentrations were measured in all subject groups prior to the ingestion of juices or water. There was no significant difference in baseline plasma cGMP concentrations among three subject groups (Table 1). The YBR juice ingestion caused a slight but significant (*P* < 0.05) increase of plasma cGMP over the baseline value at 2.5 h and then it declined towards the baseline at 5 h (Fig. 5). In comparison with the control group, the plasma cGMP concentration at 2.5 h after the ingestion was significantly higher in YBR group. However, there was no significant change of the plasma cGMP after the ingestion of GF juice (Fig. 5).

### Systemic nitrite concentrations vs. plasma cGMP concentrations

**Fig. 6:**
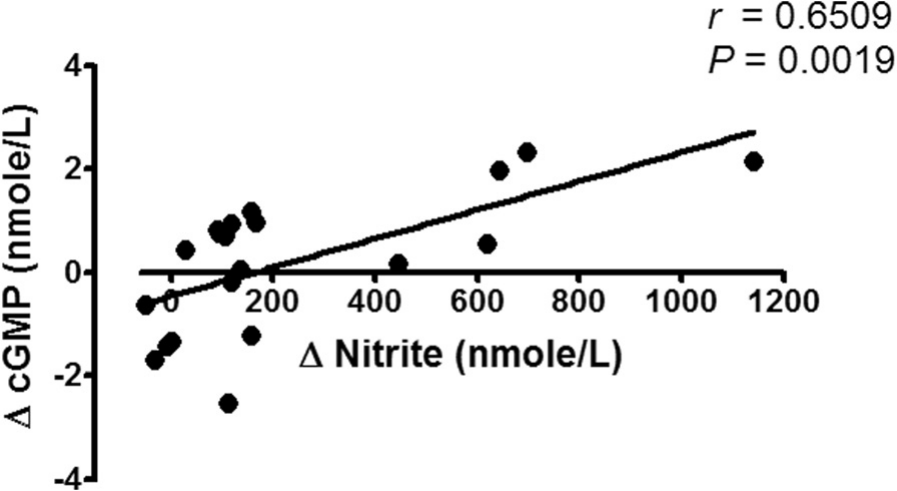
The association between plasma cGMP and plasma nitrite concentrations. *Legend:* A positive correlation between the change of plasma cGMP concentrations vs. the change of circulating nitrite concentrations in healthy volunteers following YBR juice ingestion (*n* = 10)

After the YBR juice ingestion, a significant and positive correlation (*P* < 0.05) between the increase of systemic nitrite concentrations and the increase of plasma cGMP concentrations was evident (Fig. 6). However, no correlation between these two variables was found after the ingestion of GF juice (data not shown).

### Plasma cGMP concentrations *vs*. collagen-induced platelet aggregation

**Fig. 7:**
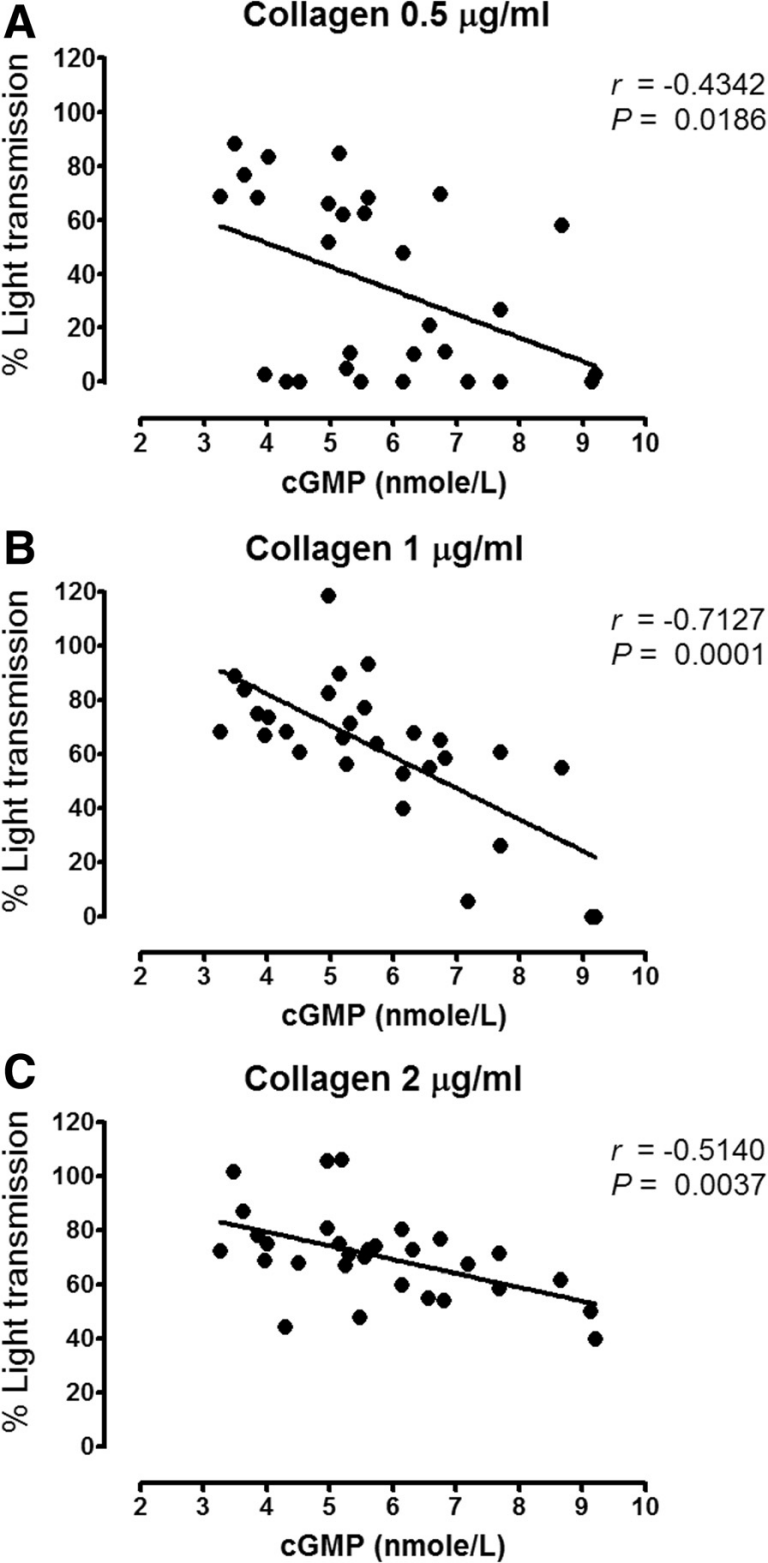
The association between plasma cGMP concentrations and collagen-induced platelet aggregation. *Legend:* The negative correlation between plasma cGMP concentrations and the maximal extent of platelet aggregation (% Light transmission) induced by (**a**) 0.5 μg/ml collagen (**b**) 1 μg/ml collagen and (**c**) 2 μg/ml of collagen in YBR juice ingested volunteers (*n* = 10)

Correlations between systemic nitrate, systemic nitrite, or plasma cGMP concentrations *vs*. the magnitude of ex vivo collagen-induced platelet aggregation were examined. Results showed a significant and negative correlation (*P* < 0.05) between plasma cGMP concentrations and the maximum extent of platelet aggregation induced by varying concentrations of collagen – 0.5 μg/ml (Fig. 7a), 1 μg/ml (Fig. 7b) and 2 μg/ml (Fig. 7c), in YBR juice group. No correlation between these two variables was found in GF juice group (data not shown).

### Effects on SBP, DBP and HR

**Fig. 8:**
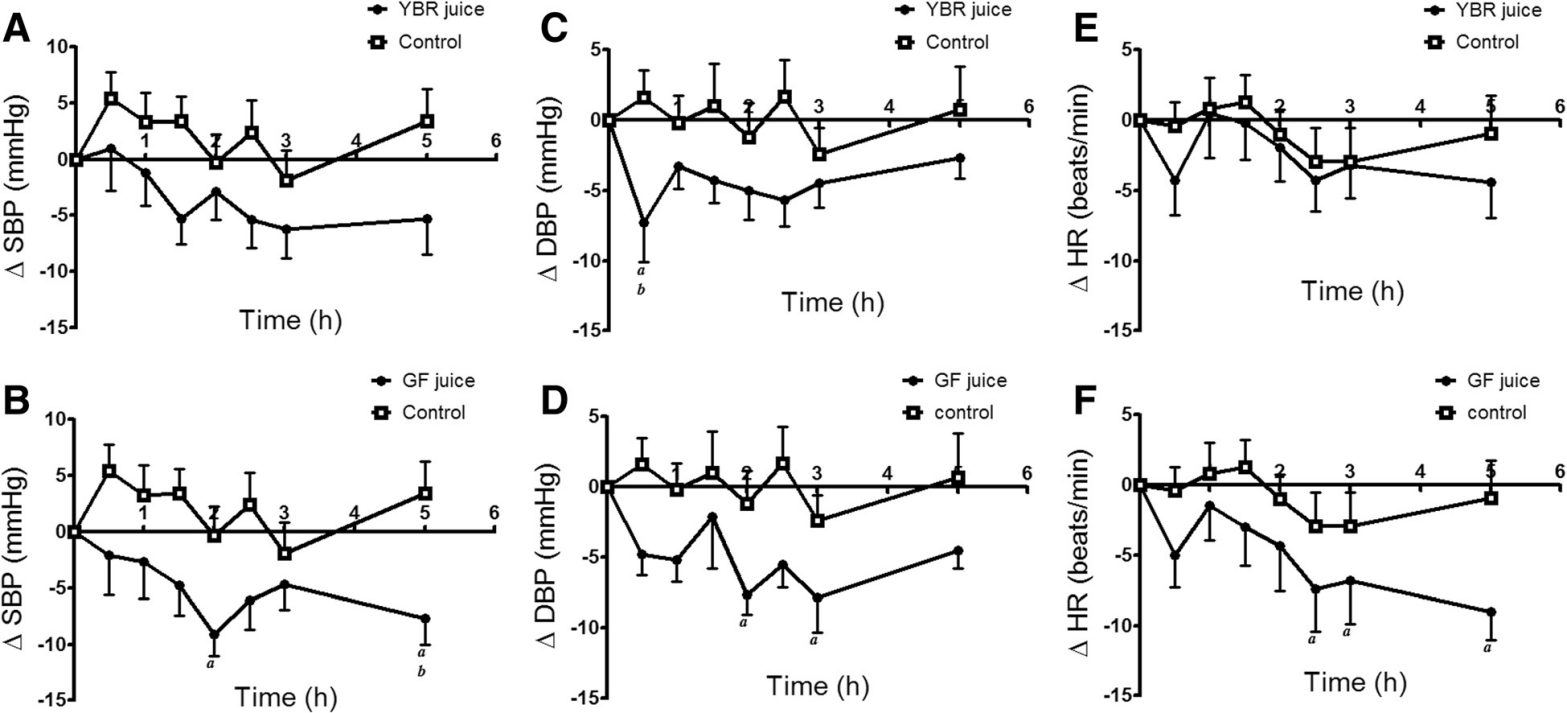
Acute cardiovascular changes after the juice ingestion. *Legend:* Effects of YBR juice ingestion on - SBP (**a**), DBP (**c**) and HR (**e**); effects of GF juice ingestion on - SBP (**b**), DBP (**d**) and HR (**f**) as compared to the control. Data are expressed as mean ± SEM from 10 subjects in each group. ^*a*^ *P* < 0.05 Dunnett’s posttest compared to the baseline, ^*b*^ *P* < 0.05 Bonferroni posttest *vs.* the control

Effects of YBR and GF juice consumption on SBP, DBP and HR were observed in healthy volunteers. At the baseline, there was no difference in means (± SEM) of SBP, DBP and HR among healthy subjects in all groups (Table 2). Comparing with the water control, a significant reduction (*P* < 0.05) of DBP was apparent only at 0.5 h after YBR juice intake but not at other time points (Fig. 8c). YBR juice ingestion caused no change in SBP (Fig. 8a) or HR (Fig. 8e) as compared to baseline values. On the contrary, the ingestion of GF juice significantly (*P* < 0.05) reduced SBP (Fig. 8b), DBP (Fig. 8d) and HR (Fig. 8f) at many time points as compared to baseline values. However, only SBP reduction at 5 h after GF juice intake was significantly (*P* < 0.05) different from that of control (Fig. 8b).

**Table 2:** Time courses of SBP, DBP and HR values before and after YBR, GF, or water ingestion

Time (h)	SBP (mmHg)			DBP (mmHg)			HR (beats/min)		
	Control	YBR	GF	Control	YBR	GF	Control	YBR	GF
0	103.10	111.90	116.20	69.30	72.00	73.22	76.70	74.10	77.11
	±3.38	±3.25	±2.71	±2.82	±2.46	±1.95	±3.07	±3.64	±3.36
0.5	108.50	112.90	114.10	70.90	64.70^*a,b*^	68.44	75.30	69.80	72.44
	±3.58	±2.98	±3.25	±2.86	±4.15	±2.29	±2.77	±4.55	±1.58
1	106.40	110.70	113.50	69.10	68.70	68.00	77.50	74.60	76.78
	±2.84	±2.98	±2.64	±2.24	±1.89	±1.61	±3.60	±4.99	±3.53
1.5	106.50	106.60	111.40	70.30	67.70	71.11	77.00	73.90	75.11
	±2.81	±3.20	±2.07	±3.09	±2.65	±3.14	±3.43	±4.10	±1.52
2	102.80	109.00	107.10	68.10	67.00	65.56^*a*^	75.70	72.20	74.33
	±3.61	±3.62	±3.04	±2.84	±1.61	±2.50	±2.89	±4.04	±2.43
2.5	105.50	106.50	110.10^*a*^	71.00	66.30	67.67	73.80	69.80	71.56^*a*^
	±2.24	±3.13	±3.21	±2.69	±2.51	±1.77	±2.69	±3.94	±2.65
3	101.20	105.70	111.50	65.90	67.50	65.33^*a*^	73.80	70.90	72.11^*a*^
	±1.65	±2.28	±2.13	±1.88	±3.08	±1.43	±3.50	±4.04	±2.17
5	106.50	106.60	108.50^*a,b*^	70.00	69.30	68.67	74.80	69.70	69.78^*a*^
	±2.32	±1.99	±2.77	±2.54	±2.13	±1.41	±3.99	±4.07	±2.15

Data are expressed as mean ± SEM from 10 subjects in each group. ^*a*^ *P* < 0.05 Dunnett’s posttest compared to the baseline, ^*b*^ *P* < 0.05 Bonferroni posttest *vs.* the control

## Discussion

Following the ingestion of YBR juice but not GF juice, systemic nitrate concentrations increased rapidly (within 30 min, peaking at 1.5 h). Our results were in accordance with previous studies which showed that dietary nitrate was readily absorbed from the stomach and proximal intestine into the blood [13, 14]. Systemic nitrite concentrations, however, increased slowly (within 1.5 h), peaked at 2.5–3 h and returned to the baseline value at 5 h. Since ingested YBR juice contains substantial amount of nitrate with undetectable quantity of nitrite, it was unlikely that systemic nitrite originated from absorbed dietary nitrite. In addition, following the ingestion of YBR juice, levels of plasma nitrite were elevated later than those of plasma nitrate (1.5 h *vs*. 30 min), reflecting the bioconversion of nitrate to nitrite in the body. Our observation was consistent with studies using inorganic nitrate and dietary nitrate (beet root juice) [13, 14]. Previous studies showed that systemic nitrite can be derived from the reduction of nitrate, originated either from NO or diet, to nitrite by two routes. Firstly, nitrate can be reduced to nitrite by commensal facultative anaerobic bacteria present in the oral cavity (i.e. mouth) and possibly in the gastrointestinal tract [36–38]. In a recent cross-over designed study with seven healthy human volunteers, rinsing the mouth with an antibacterial mouth wash has been shown to abolish conversion of nitrate to nitrite in the saliva, and markedly reduce increase in plasma nitrite following a dietary nitrate load [38]. It is thus suggested that oral bacteria play a crucial role in the reduction of nitrate to nitrite and contribute to systemic nitrite levels. Secondly, the other possibility for systemic nitrate reduction to nitrite is nitrate reductase enzymes present in tissues such as lung, liver, heart, and kidney [39]. It was demonstrated that mammalian tissues reduce inorganic nitrate to nitrite and this effect was significantly inhibited by allopurinol, suggesting that molybdenum-based oxidoreductase enzymes, such as xanthine oxidase, present in mammalian tissues may catalyze, at least in part, the reduction of nitrate to nitrite and contribute to systemic nitrite levels. In harmony with previous studies, the present study suggested that following the ingestion of YBR juice the nitrate content in YBR juice was absorbed and was further converted to nitrite in the body.

In this study, we have shown that both YBR and GF juices attenuated ex vivo collagen-induced platelet aggregation. However, only the inhibition by YBR juice at 2.5 h after the ingestion was concurrent with the peaking of systemic nitrite concentrations. Moreover, at 5 h after YBR juice ingestion, both systemic nitrite concentrations and collagen-induced platelet aggregation were simultaneously returned to baseline values. Supplementation with 2 mmole/L potassium nitrate has been demonstrated to inhibit platelet aggregation [40]. Dietary supplement with nitrate (beetroot juice) has been shown to inhibit ex vivo platelet aggregation in healthy volunteers [14]. Moreover, nitrite was shown to inhibit platelet aggregation in a cat model [41]. Our results suggest that systemic nitrite derived from dietary nitrate in YBR juice may involve in the mechanism of platelet inhibition.

Recently, Sihirun et al. [42] showed that nitrite itself did not directly inhibit platelet aggregation in vitro and suggested that nitrite might be further converted to NO which attenuated platelet aggregation. Cosby et al. [43] demonstrated increased NO formation in the blood, as measured by the rate of formation of ironnitrosylated hemoglobin (HbNO) during nitrite infusion at rest and under exercise, suggesting that nitrite represents a major bioavailable pool of NO. Recently, it was shown that inorganic nitrate ingestion in males elevated platelet cGMP levels, implicating a role of NO in the anti-aggregating effect of nitrate in vivo [44]. Accordingly, we demonstrated that following YBR juice ingestion, similar concentration-time profiles of systemic nitrite and plasma cGMP were apparent. In addition, a positive correlation (r = 0.6509, *P* < 0.05) between the extent of increases in plasma nitrite from the baseline and the extent of increases in plasma cGMP from the baseline was evident. Nitrate was previously shown to increase the plasma cGMP [45]. Arteriolar cGMP production has been used to assess the activation of soluble guanylyl cyclase in vivo [45]. Many investigators adopted nitrate and plasma cGMP measurements as a reliable indicator of NO amplification during physiological [4, 7] and pharmacological stimulations [5, 8]. Moreover, a previous study showed that collagen-induced platelet adhesion and aggregation were inhibited by NO donor *S*-nitrosoglutathione (GSNO) [46]. Our results also showed the negative correlation between plasma cGMP concentrations and the maximal extent of ex vivo platelet aggregation induced by varying concentrations of collagen (0.5–2 μg/ml) in YBR juice group. During inhalation of NO, increased plasma cGMP concentrations and inhibition of platelet aggregation have been previously reported [47, 48]. Therefore, results of the present study in conjunction with previous studies suggested that following YBR ingestion, systemic nitrite that converted from systemic nitrate was further converted to NO which attenuated platelet responses to collagen stimulation. On the contrary, our results demonstrated that YBR juice intake did not inhibit ADP-induced platelet aggregation.

The ingestion of GF juice also attenuated collagen-induced but not ADP-induced platelet aggregation. However, our results showed that the inhibition of collagen-induced platelet aggregation by GF was not associated with systemic nitrate and nitrite concentrations. Guava is a flavonoid-rich fruit [49, 50], and flavonoid compounds existed in guava fruit were reported including myricetin, apigenin, leucocyanidin, quercetin, quercetin-3-α-L arabinofuranoside, quercetin 3-β-D glucoside, quercertin 3-β-galactoside, and quercitrin [50, 51]. Hubbard and coworkers [52] proposed that the ingestion of quercetin-4-o-β-d-glucoside inhibited collagen-induced platelet aggregation via the reduction in phosphorylation of tyrosine kinase Syk and phospholipase Cγ2, which are signaling components of the platelet glycoprotein VI collagen receptor. In addition, some studies suggested possible mechanisms by which flavonoids exert their antiplatelet property; lowering intracellular Ca^2+^ levels, alteration in the metabolism of cAMP and thromboxane A_2_ [53, 54]. It is likely that flavonoids in GF juice might be responsible for the platelet inhibitory action, a speculation that requires further investigations.

Webb and coworkers [14] demonstrated that the ingestion of nitrate load (beetroot juice) reduced both systolic and diastolic blood pressure in healthy human volunteers, an effect that correlated with the peak increase in plasma nitrite concentrations. Our study showed that YBR juice reduced DBP only at 0.5 h after the ingestion and this response showed no association with both nitrate and nitrite concentrations. Lacking of cardiovascular effects of YBR juice may be due to relatively low systemic concentrations of both nitrate and nitrite after YBR ingestion as compared to those of beet root juice. Peak concentrations of systemic nitrate and nitrite after YBR juice ingestion were 150 μmole/L and 0.4 μmole/L, respectively, and those after beetroot juice ingestion were 400 μmole/L for systemic nitrate and 0.6 μmole/L for systemic nitrite. In general, it is rather difficult to see changes of cardiovascular responses induced by low concentrations of these substances in healthy volunteers. However, these variables may be altered by emotional changes, the position of subjects, as well as pathological conditions. Our results showed that GF juice ingestion affected all cardiovascular variables measured. The effect of GF juice on SBP in the present study is supported by a previous study which showed the antihypertensive effect of pink guava puree in spontaneous hypertensive rats [25]. Vasodilator and antioxidant actions exerted by the guava extract have also been reported [24]. In patients with essential hypertension, guava extracts supplemented for twelve weeks significantly decreased SBP with a significant increase in HDL [55]. Our results also demonstrated the cardiac depressive effect of GF juice intake. Similarly, guava leaf extract was previously demonstrated to have cardiac depressant activities in the cardiac tissue [27] and animal model [56]. Overall, the apparent limitation of our study is using a relatively small number of subjects per group which might lead to the arbitrary generalization of study outcome.

## Conclusion

The present study showed, for the first time, the acute platelet inhibitory effect of YBR juice and GF juice ingestion in healthy volunteers. YBR juice contained a substantial amount of dietary nitrate which, following ingestion, was absorbed and further converted to nitrite in the body. Systemic nitrite might be further converted to NO which attenuated platelet responses to collagen stimulation following YBR juice ingestion. In addition, the ingestion of YBR juice reduced only DBP while the ingestion of GF juice reduced SBP, DBP and HR. As our concern on primary prevention, the consumption of YBR and GF juices might promote public cardiovascular health through their roles in platelet inhibition and blood pressure lowering. Further works are needed to fully elucidate the molecular mechanism of actions of YBR and GF juices in several physiological processes in order to prove their prophylactic and therapeutic potentials.

## Abbreviations

cGMP, cyclic guanosine monophosphate; DBP, diastolic blood pressure; GF, guava fruit; HR, heart rate; NO, nitric oxide; SBP, systolic blood pressure; YBR, yam bean root
